# Summer season temperature-humidity index threshold for infrared thermography in Hanwoo (*Bos taurus coreanae*) heifers

**DOI:** 10.5713/ajas.19.0762

**Published:** 2020-01-13

**Authors:** Na Yeon Kim, Sang Ho Moon, Seong Jin Kim, Eun Kyung Kim, Mirae Oh, Yujiao Tang, Se Young Jang

**Affiliations:** 1 Division of Food Bio Science, Konkuk University, Chungju 27478, Korea; 2 National Institute of Animal Science, RDA, Sunghwan 31000, Korea; 3 Institute of Livestock Environmental Management, Sejong 30127, Korea

**Keywords:** Temperature Humidity Index (THI), Body Surface Temperature, Hanwoo, Animal Welfare, Heat Stress

## Abstract

**Objective:**

The study sought to estimate the relationship between body surface temperature (BST) and temperature humidity index (THI) and to present the validity of THI as a heat stress index in the field.

**Methods:**

Eight Hanwoo heifers (20 to 32 month) were examined in a field trial, with a space allowance of 10 m^2^ per head. The BST was measured using an infrared thermographic camera. The BST of five body regions (eyes, hindquarters, nose, part of horns, and ears), ambient temperature (AT), and relative humidity (RH) were measured 7 times daily (07, 09, 11, 13, 15, 17, and 19 h) during each season with three replicates.

**Results:**

The THI ranged 34.0 to 56.9 during spring (AT, −1.0°C to 13.4°C), 75.1 to 84.7 during summer (AT, 24.9°C to 33.6°C), 55.8 to 70.9 during autumn (AT, 13.0°C to 26.0°C) and 17.5 to 39.2 during winter (AT, −10.4°C to 1.0°C). In the regression analysis, the coefficient of determination (R^2^) between THI and BST was 0.88, 0.72, 0.83, 0.86, and 0.85 for the eyes, hindquarters, nose, part of horn, and ears area, respectively. This indicates that BST has a strong correlation with AT and RH. Expression equations were estimated as Y (THI) = 31.54+0.1085X (BST of eyes) and Y (THI) = 30.48+0.1147X (BST of hindquarters) by simple linear regression analysis in this experiment.

**Conclusion:**

Consequently, the upper bound for heat stress estimation can be specified ranging from THI of 65 (eyes) to 70 (hindquarters). From this we can expect a precise feeding system for Korean native cattle in the field.

## INTRODUCTION

Precise monitoring of the physiological status of farm animals is one of the most important issues in the livestock industry [1,2]. In general, animal health management animals is determined by body temperature, heart rate and blood analysis [3]. However, such measurements are disadvantageous because they require physical contact [4], and it is also difficult to measure the temperature of large animals. Schaefer et al [5] suggested that the infrared thermographic (IRT) technique could be used effectively for non-invasive monitoring of body surface temperature (BST) in the field. The IRT technique is fast and simple McManus et al [4] and can be used for early diagnostic detection of diseases [6]. IRT can also be applied to multiple animals at the same time. Colak et al [7] have reported that the IRT technique can provide sensitive information concerning mammary changes for mastitis diagnosis. According to a report using a thermal camera, BST measurement is a useful method for detecting the health status of livestock. However, many studies have focused on direct diagnosis of diseases. There is a continuing need for research on the impact of environmental factors on livestock health. Korea has four distinct seasons with a wide temperature range [8]. More particularly, the summer in Korea is characterized by high temperatures and humidity which have a large influence on the livestock industry [9–11]. In principle, heat stress can decrease feed intake [12] and thus the productivity of animals [13]. In addition, environmental determinants might have different influences on different species. Hanwoo (*Bos Taurus coreanae*), the native Korean cattle used in the current study is the main breed for meat production in Korea. For feeding standards, the upper bound critical temperature was determined to be 30°C [14], but there has been no definitive research on the matter. This is compounded by the fact that the sensible temperature can differ from the ambient temperature (AT), because sensible temperature depends on a variety of environmental factors such as air temperature, relative humidity (RH), wind velocity, heat radiation, and rainfall [15]. Among them, the AT is the most dominant factor that influences the heat environment.

The current study used air temperature and humidity to calculate the temperature humidity index (THI). The THI can be used as an indicator of heat stress [16–18]. However, further experiments are required, because little information exists on the variation of THI and the relationship between THI and BST. Depending on environmental factors, BST may change, and an analysis of the correlation between animal-based BST and the external factor THI is needed. This study therefore investigates the relationship between BST and THI and considers the validity of THI as a stress index in the field. To support decision-making, five distinct areas will be comparatively analyzed using IRT.

## MATERIALS AND METHODS

### Animals and management

This experiment was implemented under the approval of the Institutional Animal Care and Use Committee of Konkuk University (IACUC No. Ku 12097).

This study was performed at the experimental farm in Gyeonggi-do, South Korea, from January through December 2014. Heifers were managed according to the guidelines set by the Hanwoo Feeding Standard [19]. Eight Hanwoo (*Bos taurus coreanae*, 20 to 32 month) heifers were individually housed in a pen that provided a 10 m_2_ of -space per head. The pen was covered with a 4.5-m roof that induced ventilation and included a winch curtain, that prevented the entry of cold wind in the winter. The curtain was mostly open, save when there was a strong cold winter wind. Each heifer was offered total mixed ration (TMR), per the Hanwoo feeding standard (Table 1). The animals had free access to feed and water.

### Sampling of body surface temperature thermography and temperature humidity index

**Figure 1:**
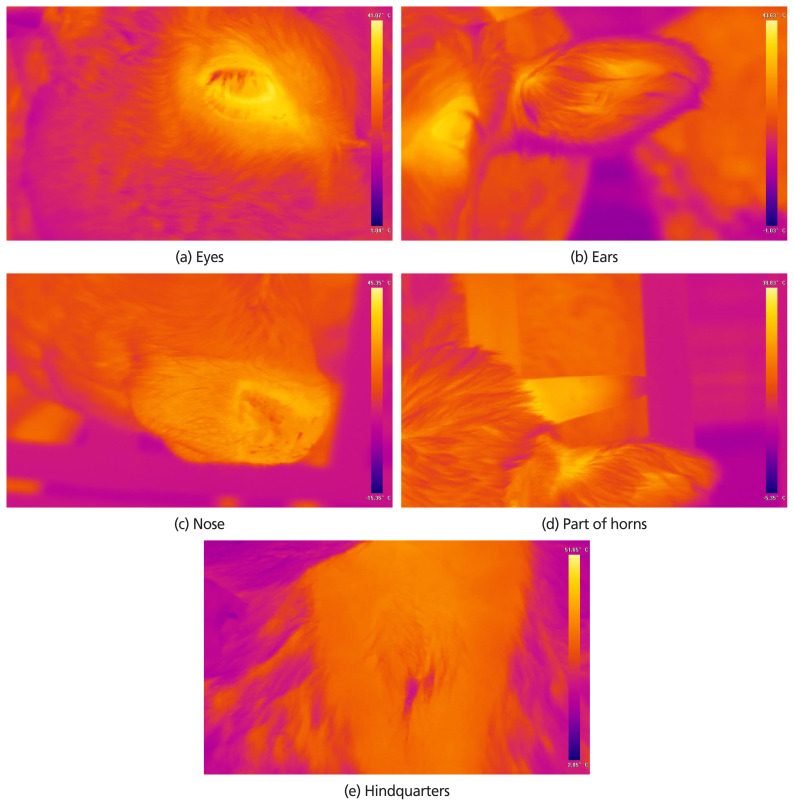
Infrared thermal image samples according to the five areas. (a) Body temperature around the eyes, (b) body temperature around the ear, (c) body temperature around the nose, (d) body temperature around the part of horns, (e) body temperature around the hindquarters. The blue area is at low temperature and the red area is at high temperatures.

Data collection for thermal images was initiated at 07:00 a.m. Before temperature measurement, all heifers were held at a head control bar for short distance. An infrared thermal imaging camera CX-320U (COX, Taejeon, Korea) was used for BST measurements. Samples of BST that were collected from a distance greater than 3 m from the camera were excluded due to the sensitivity of the camera, which works precisely within the 3-m range. Five distinct areas were measured to evaluate BST, the eyes, nose, part of horns, ears and hindquarters (Figure 1 for sample images). The IRT images were collected 7 times daily at 07, 09, 11, 13, 15, 17, and 19 hours. Core body temperature (CBT) was recorded by digital thermometer inserted into the rectum. AT and RH were recorded at the same time as IRT sampling. The THI was calculated using the following equation: THI = (1.8 temperature °C+ 32) −(0.55–0.0055×humidity)×(1.8 temperature °C–26) following [18].

### Statistical analysis

Data were expressed as means and standard deviation and were statistically analyzed with Tukey multiple range tests using the SPSS package (ver. 23) general linear models procedure. Regression analysis was performed between the BST and THI. This is based on an assumption that the core temperature of the animal is not varying significantly. The BST areas were used for the dependent variable and THI was used the independent variable. Regression coefficients were estimated for changes of THI. Regression equations were assumed for THI and BST (eyes, hindquarters, nose, part of horn, and ears).

## RESULTS

### Variation of body temperature according to seasonal changes

**Figure 2:**
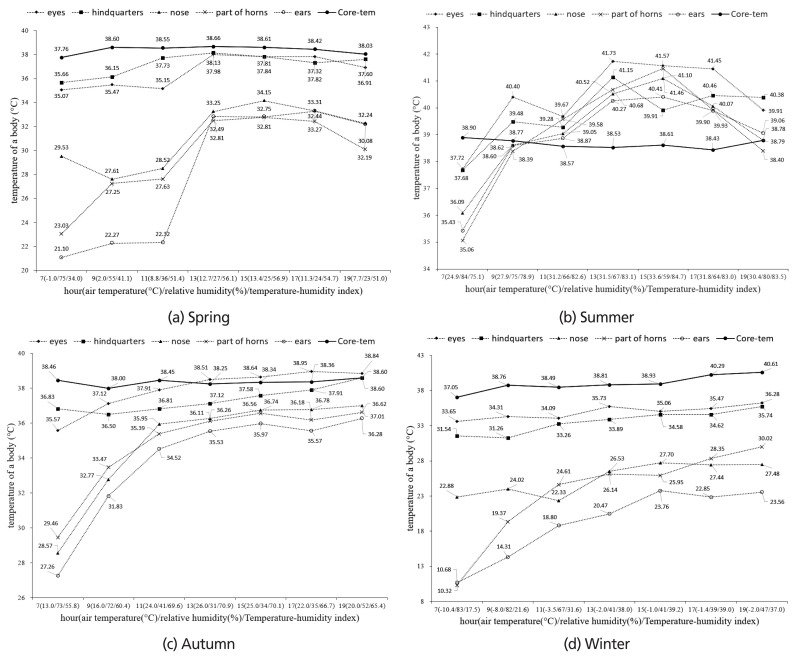
Seasonal daily body surface temperature changes in Hanwoo heifers. (a) Spring, (b) summer, (c) autumn, (d) winter. Eyes, body temperature around the eyes; hindquarters, body temperature around the hindquarters; nose, body temperature around the nose; part of horns, body temperature around the part of horns; ears, body temperature around the ear; core-tem, core body temperature was recorded by digital thermometer inserted into the rectum.

The BST and CBT of changes are shown in Figure 2. The AT in spring was variable, ranging from −1°C to 13.4°C. The temperature changes in the eyes and hindquarter areas were stable on the whole. There was a definite change from 11 to 13 hour in the nose, part of horns and ears areas. At this time, THI increased from 51.4 to 56.1. The temperature varied from 24.9°C to 33.6°C in summer. The AT reached a record high (33.6°C) at 15 h. Characteristically, the BST level was higher than CBT at 11 h in all areas. The highest BST recorded was 41.73°C at 13 h for the eyes area. The THI ranged from 78.9 to 82.6 between the hours of 9 and 11. At the same time, the BST was remained higher than CBT. The AT varied from 13.0°C to 26.0°C in autumn. The BST of the eyes was higher than CBT at 13 hour and the THI level was 70.9. After 13 h, the BST of the eyes consistently showed higher measurements than CBT. There were stable changes in the eyes and hindquarter areas. The temperature varied from −1.0°C to −10.4°C in winter. Overall, the BST was lower than in the other seasons. The temperature changes in the eyes and hindquarter areas were stable on the whole and were similar to CBT changes. There were dramatic changes in BST in the part of horns and ears areas.

The comparison for the BST is presented in Figure 2. The data were analyzed for each hour. There were significant (p< 0.05) differences among BST measurements in spring. The BST was variable, ranging from 35.07°C to 37.98°C (eyes) or 35.66°C to 38.13°C (hindquarters) on average. Compared with CBT, there was an average difference of 2 degrees between CBT and BST for the eyes and hindquarter areas. The BST of the eyes and hindquarter was higher than other areas, and there was a variable gap between the other areas. There were significant (p<0.05) differences among the BST measurements, but BST was very irregular in all areas. There was no difference at 11 hour among all areas. There were significant (p<0.05) differences among the BST in autumn. The BST was variable ranging from 35.57°C to 38.95°C (eyes) or 36.50°C to 38.60°C (hindquarters) on average. Depending on THI, there was large changes in some areas, up to 8.44°C (nose), 7.16 (part of horns), and 9.02°C (ears). There were significant (p<0.05) differences among the BST in winter. The BST of the eyes and hindquarter areas were higher than other areas throughout. The part of horns showed the lowest temperature (10.32°C) at 07 hour.

### Prediction model for relationship between temperature humidity index and body surface temperature of eyes

**Figure 3:**
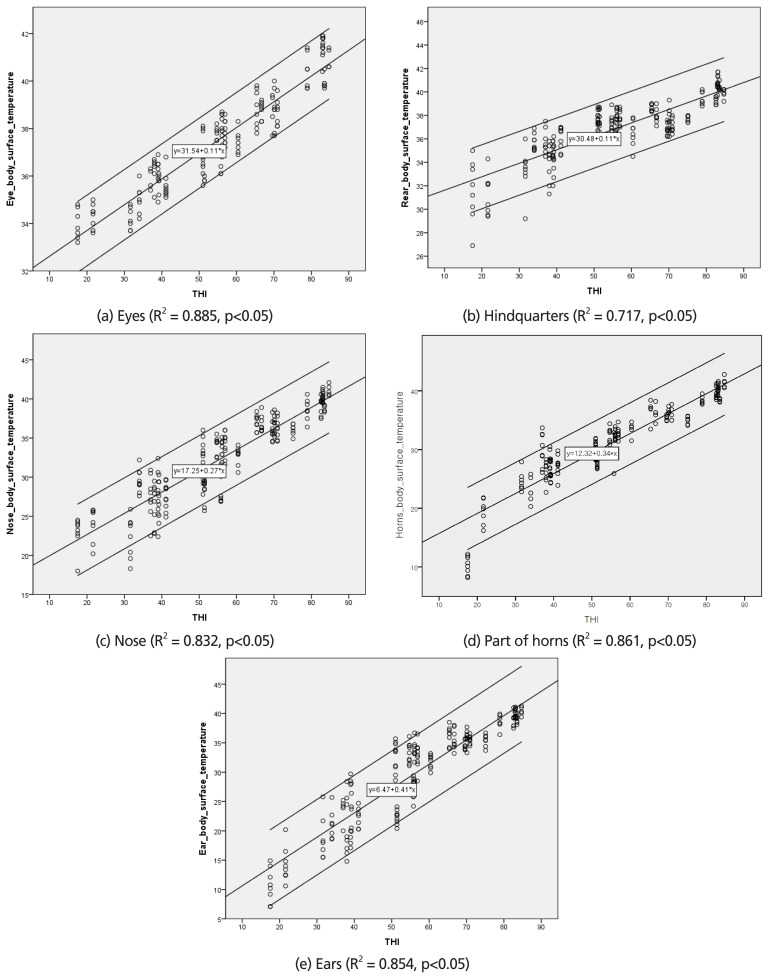
Scatter plot and regression model of temperature-humidity index (THI) (Ravagnolo et al [18]) and body surface temperature in Hanwoo heifers. Data were expressed as means and standard deviation and were statistically analyzed with Tukey multiple range tests using the SPSS package (ver. 23) general linear models procedure (n = 208).

Figure 3 shows a summary of the basic statistics of the regression analysis data set. We used statistical analysis to perform simple regression to evaluate the effect of THI on the BST of all scanned body areas. For all areas, THI significantly affected the BST (p<0.05), and the higher the THI, the greater the BST of the body area in question. For regression related to the BST of the eyes, the expression equations were estimated as Y (THI) = 31.54+0.1085X (BST of eyes), the BST of the eyes was explained by THI R^2^ = 88.5%. For the hindquarters, the expression equations were estimated as Y (THI) = 30.48+ 0.1147X (BST of hindquarters), and BST of the hindquarters was explained by THI R^2^ = 71.7%. For the nose, the expression equations were estimated as Y (THI) = 17.24+0.2709X (BST of nose), and the BST of the nose was explained by THI R^2^ = 83.2%. For the part of horns, the expression equations were estimated as Y (THI) = 12.31+0.34X (BST of part of horns), and the BST of the part of horns was explained by THI R^2^ = 86.1%. For the ears, the expression equations were estimated as Y (THI) = 6.46+0.4149X (BST of ears), and the BST of the ears was explained by THI R^2^ = 85.4%.

## DISCUSSION

Body temperature is one of the most common indicators used for the diagnosis of illness in livestock and has been used in the measurement of the physiological status of animals [20]. As can be seen in Figure 2, the BST was lower than the CBT in all seasons except for summer. Normally, the BST should be less than the CBT as a result of heat transfer and blood flow [21]. In general, the core temperature averages 38.5°C for Hanwoo cattle [22]. Because cows are homeothermic, they should be kept in a condition of homeostasis [23]. THI and BST are not statistically clearly related, but because the core temperature of livestock is constant, BST is independently affected by THI. In the current study, the BST was higher than the CBT only in summer, and the average CBT was 38.6°C. Conditions in which the BST was higher than CBT are an abnormal observation. Recent findings indicate that CBT can be used as an indicator of stress when the values of the BST are higher than CBT [24], and the abnormal values indicate that only summer is especially worthy of notice to estimate specific THI.

Previous studies have indicated that THI is generally used to estimate the effect of climatic conditions [18,25]. The value for the THI can also be used to estimate the stress situation, for example, Ravagnolo et al [18] reported that milking frequency declines when THI exceeds 72. Allen et al [16] reported that standing behavior relates to heat stress and is affected when THI reaches 68. Bernabucci et al [26] reported that milk yield declined when THI ranged from 65 to 76. From these reports and the findings of the present study, it appears that the THI could be useful for estimating of heat stress situations.

In the regression analysis, the coefficients of determination (R^2^) between THI and BST were 0.88, 0.72, 0.83, 0.86 and 0.85 for eyes, hindquarter, nose, part of horns, and ears areas, respectively (p<0.05). These results agreed with those of Salles et al [24], who reported that IRT is strongly correlated with BST, suggesting that BST has a strong correlation with environmental factors.

Expected values are dependent on the measurement areas. Researchers need to determine which body measurements are significant in estimating the physiological status of an animal [3]. In the current study, the BST data collected at different parts of the body are available for comparison, and the data showed that the BST values were significantly different (p<0.05) at each area (Figure 2), with the temperature range being rather large. However, the eyes and hindquarter areas, as well as other areas, remain meaningful for the measurement. In previous studies, other researchers [3,27] reported that eyes region of steers is the most suitable part for measurement. In the current study, the temperature measurements at the eyes and hindquarter areas were higher than other areas and the temperature changes at the eyes and hindquarters had greater stability throughout the year.

The THI can be presented as an indicator to contribute to the stress suggestion in the field through the estimation of regression equations. As mentioned above, the expression equations were estimated as Y (THI) = 31.54+0.1085X (BST of eyes) and Y (THI) = 30.48+0.1147X (BST of hindquarters) through simple linear regression. The upper bound for heat stress estimation can be specified for the range from THI 65 (eyes) to 70 (hindquarters, Figure 2). Similar results were obtained in other studies [16,18,26].

Inserting a thermometer directly into the rectum is an inefficient method of measuring temperature in livestock, and the IRT technique is both more efficient and easier. IRT can also allow the comparison of many cows at the same time and abnormal livestock can be quickly identified [20,28]. IRT therefore has many potential advantages, but it should be noted that IRT can be influenced by a multiplicity of different factors, including room temperature based on camera position. This should be kept in mind as feeding environments are irregular and the housing systems for livestock are diverse [21]. The results of this investigation may, however, still provide a better understanding of the associations between BST measured at different points on the animal, as well as the relationship between these areas and environmental variables.

## CONCLUSION

The BST measurements using IRT may be one of the most effective tools for precision heifer farming when sufficient information concerning the characteristics of BST and environmental factors have been collected. Among the body regions studied, IRT temperature showed the highest correlation with surface temperature, and the eyes and hindquarter regions of heifers were identified as the most stable areas for IRT measurements. In hot climates, farm managers should consider weather conditions to reduce heat stress in the field. Based on the results of this study the upper bound for heat stress estimation appears to be within the range of THI 65 (eyes) to 70 (hindquarters). Based on these finding, more precise feeding and care systems for Korean native cattle in the field can be expected.

## Figures and Tables

**Table 1:** Ingredient composition of experimental diet

Ingredient	Composition (% of dry matter)
Perennial ryegrass	6.0
Annual ryegrass	3.0
Alfalfa hay	2.0
Klein grass	1.0
Corn, flaked	15.0
Wheat meal	26.5
Soybean meal	15.0
Corn gluten meal	8.0
Coconut meal	9.1
Lupin, flaked	2.0
Whole cottonseed	2.0
Molasses	5.0
Yeast culture	2.0
Limestone	2.5
Salt	0.6
Mineral	0.3
